# Review of Problems of Living: Perspectives from Philosophy, Psychiatry and Cognitive-Affective Science

**DOI:** 10.4102/sajpsychiatry.v27i0.1834

**Published:** 2021-12-14

**Authors:** Randolph M. Nesse

**Affiliations:** 1 Department of Psychiatry, University of Michigan, Ann Arbor, United States of America; 2 Department of Life Sciences, Arizona State University, Tempe, United States of America

Like many psychiatrists-to-be, I once looked to philosophy for answers to the big questions. What is good? Why is life full of suffering? Does life have meaning? The possibility of answers made the idea of becoming a philosopher appealing. However, the philosophy courses I took were mainly close reading of historical backgrounds and arcane arguments about definitions; finding out what is true seemed to be a low priority. So, like many others, I drifted away to psychology and biology.

Dan Stein persisted. His book reflects a lifetime of reading diverse philosophers deeply, all the whilst keeping his focus on the big questions and how philosophical answers can inform our understanding and treatment of mental disorders. For those of us who gave up that quest, his book allows us to imagine what our lives might have been like if we had devoted ourselves equally to philosophy and psychiatry for decades … and if we had been much smarter.

The book’s title, *Problems of Living*, signals correctly that it is approachable and of special interest for anyone interested in human mental life. However, it might more accurately have been titled, ‘A thoughtful synthesis of what philosophers and cognitive and neuroscientists have said about the big questions relevant to human life and mental disorders’. It is really that comprehensive.

The book is organised by seven big questions, with a preview describing why they are important for psychiatry and the author’s approach to integrating diverse perspectives, as exemplified by the tables that summarise each chapter. In Chapter 1, a table and the text distinguish classical, critical and integrative perspectives on philosophy of science, philosophy of language, natural kinds and so on. The very idea of providing just a few words in neat boxes to summarise centuries of controversies is audacious, but it works. Alas, I fear they will also make it easy for teachers to write test questions that deaden students’ enthusiasm. However, the book is so fascinating that it would make an ideal text for seminars on philosophy, in general, as well as more specialised seminars on philosophy and psychiatry.

Most pages are half taken up with footnotes. They seem daunting and boring until you read them and discover that they contain anecdotes, quips, quotes and opinions that are as interesting as the text itself. For instance, footnote 11 describes a seminar in which Wittgenstein waves a fireplace poker to emphasise his demand that Karl Popper state a moral rule. At which point Popper says, ‘not to threaten visiting lecturers with pokers’. Whereupon Wittgenstein stalks out of the room.

Chapter 2 tackles the first big question, the mind–body problem. After delving into for several years, I swore it off; if decades of debate had reached no solution, why bother? However, Stein describes and makes sense of the issues. He contrasts the views of reductionism, dualism and emergent materialism, with comments on how they inform psychoanalysis and schema therapy. He then uses the metaphor of ‘wetware’ to tie his synthesis in with modern cognitive science.

The next chapter tackles the history of the reason vs. emotion dichotomy and the genuine progress that has been made; they are now widely viewed as intimately interconnected aspects of the mental system. In footnote 22, we find gems from Montaigne, Flannery O’Connor and Susan Sontag on how writing creates thinking. The chapter concludes, as most of them do, with clinically useful implications about emotions, reason and their disorders.

Stein then tackles pleasure and happiness. It is reassuring and amusing to learn that he has ‘always been an absolute sucker for self-help books on happiness’. The chapter makes the positions of Epicureans and Stoics clear, but quickly moves on to cite the many philosophers who recognise that real happiness requires both purpose and pleasure. How wonderful, and how essential for therapists, to learn that John Stuart Mill said, ‘ask yourself if you are happy, and you will cease to be so’. We then get an up-to-date view of positive psychology, with more quotes from contemporary researchers who are sceptical that pursuing happiness can get you there.

He then turns to pain, sadness and disease, making good use of his extensive contributions about the Diagnostic and Statistical Manual (DSM) and the dilemmas surrounding psychiatric diagnosis. He values Wakefield’s ‘harmful dysfunction’ analysis but puts it in a critical context. As elsewhere, his conclusion is balanced: neither extreme optimism nor pessimism, but a realistic view that offers modest opinions and sensible advice. We are again brought back to purpose and pleasure.

The nature of good, evil and morality get a coherent treatment that is especially relevant to psychiatry. Psychopathy is exhibit A, but instead of merely reviewing the evidence, the chapter displays a commitment to understanding individuals as individuals with the conclusion that psychopathy ‘lies on a dimension with many individuals falling along a spectrum’ (p. 147). The same tendency is manifest a few pages later with the conclusion, ‘***any characterization of humans as essentially demonic or angelic is overly simplistic***’ (Bold in original). This theme continues in his discussion of the implications for psychotherapy where he concludes, ‘people may have quite different but entirely reasonable ways of achieving health, or balance, or purpose’. You can tell he has listened closely to many patients. He also brings in neuroscience, with the original observation that some people have a kind of anosognosia that prevents them from viewing their own sins. A deep consideration of new knowledge about the evolutionary origins of capacities for morality is missing, sadly, because it is crucial for understanding relationships and much that happens in psychotherapy.

How can we tell what is true? Many contemporary authors say, ‘science’, as if nothing more needs to be said. However, Table 11 provides a grand summary of classical, critical and integrative positions on the contributions from science, philosophy and humanities. I was delighted to learn that William James said, ‘philosophy is at one the most sublime and the most trivial of human pursuits. It works in the minutest crannies, and it opens out the wider vistas’ (p. 175). Table 12 goes on to compare scientism, scepticism and an integrative position for psychiatry. As elsewhere, Stein is respectful of the progress psychiatry has made, whilst not shrinking from a clear-eyed view of what we do not know.

I was eager to find answers to my existential questions in the penultimate chapter on ‘The meaning of life’. However, I had to laugh to discover that Douglas Adams’ answer, ‘42’, receives pride of place. The author’s erudition is matched only by his humour, citing Irvine Yalom’s assessment of the human dilemma as involving, ‘a being who searches for meaning and certainty in a universe that has neither’. It was satisfying to hear that ‘an integrative approach to the meaning of life emphasises that there are multiple meanings in life’.

The final chapter wraps up things by considering the richness of life and its metaphors, again with humour: ‘I particularly like versions of [*the*] metaphor of God as our father in which rebellion by the kids is acceptable’. Our lives are, he concludes, journeys, stories and narratives, in which meaning comes from meaningful action, including reading philosophy and practising psychiatry. He concludes, consistent with his emphasis on integration and balance by advising, ‘Everything in moderation, including moderation’.

This book is an antidote to the trend for proposing extreme views that generate clicks. It made me consider possible ancient examples of that tendency. Grand schemes and diametric positions garner attention. Some say mind is merely brain, others say that it is a whole separate realm. Some find meaning intrinsic to life, others find that it is constructed, if it exists at all. Many argue that moral principles are universal, others that they are merely emergent generalisations. The authors like Stein, who integrate dimetric positions, are provided less attention, but they deserve much more.

I would love to know more about Stein’s personal conclusions. Does he feel that his questions have been answered? I would guess that he finds philosophy valuable but incomplete. The enterprise of uniting it with cognitive science, neuroscience and evolutionary biology is just getting going but few have the range of expertise to advance it.

I know of no other source that so clearly and comprehensively integrates philosophy, cognitive science and neuroscience, and certainly none that use them to address problems in psychiatry. I hope this achievement will be widely appreciated, but I fear the book’s price will limit its influence. However, the price is worth it; I have already bought two copies for friends. Digital versions can be downloaded without charge from some academic libraries, to the great benefit of psychiatry, philosophy and knowledge, in general.

